# The role of species distribution models and multiple data sources in implementing dynamic ocean management

**DOI:** 10.1017/cft.2026.10041

**Published:** 2026-06-24

**Authors:** Emily C. Nazario, Helen Bailey, Rachel Rhodes, Elliott L. Hazen, Steven J. Bograd, Austin Sell, Douglas J. McCauley, Mark F. Baumgartner, Ana Širović, Briana Abrahms, Rachel Seary

**Affiliations:** 1 Ecology and Evolutionary Biology, University of California Santa Cruz, USA; 2 Blue Wave Consulting, LLC, Maryland, USA; 3 Marine Science Institute, University of California Santa Barbara, USA; 4 Institute of Marine Sciences, University of California Santa Cruz, USA; 5 Environmental Research Division, Southwest Fisheries Science Center, National Marine Fisheries Service, National Oceanic and Atmospheric Administration, USA; 6 Evans School of Public Policy and Governance, University of Washington, USA; 7 Ecology, Evolution and Marine Biology Department, University of California Santa Barbara, USA; 8 Biology Department, Woods Hole Oceanographic Institution, USA; 9 Department of Biology, Norwegian University of Science and Technology, Norway; 10 Center for Ecosystem Sentinels, Department of Biology, University of Washington, USA; 11 Durrell Institute of Conservation and Ecology, School of Natural Sciences, University of Kent, UK

**Keywords:** SDMs, WhaleWatch, blue whales, adaptive management

## Abstract

We use the Whale Safe initiative as a case study to investigate the efficacy of species distribution models (SDMs) for dynamic ocean management in a relatively data-rich context. This program combines SDMs, passive acoustic monitoring and opportunistic sightings data to help reduce whale-ship collisions off California, USA. Blue whale SDM predictions were significantly correlated with the *in situ* observations of whale presence (sightings and passive acoustic detections), suggesting that the model is a reliable source for indicating blue whale presence. We also demonstrated from this multiyear program the value of integrating multiple sources of inference (sightings, acoustics and SDMs) for achieving dynamic management. The results indicated the potential benefit of using validated SDMs in less data-rich regions to support dynamic ocean management when *in situ* real-time data sources are sporadic or unavailable.

## Graphical abstract

**Figure:**
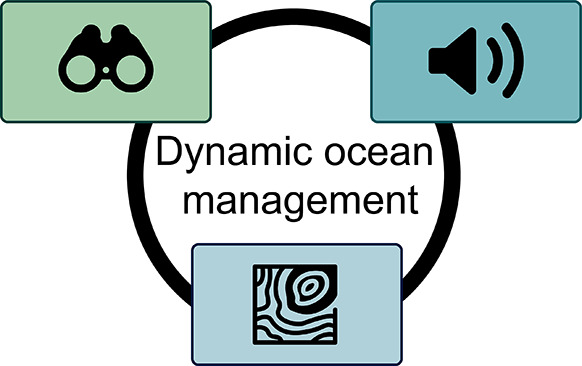

## Impact statement

Dynamic ocean management, management measures that rapidly change in space and time in response to near real-time data, is increasingly being adopted as an approach to balancing marine life protection and human activities in marine environments. This approach requires reliable near real-time biological, oceanographic or social data, which are costly to attain and maintain. Species distribution model (SDM) outputs have been utilized as a complementary source of information to predict species occurrence. Despite previous work indicating that distribution models can be used to help inform dynamic ocean management frameworks, little work has been done to assess the accuracy and reliability of operational modeled outputs compared to *in situ* surveillance data. In this article, we evaluate the value of SDMs alongside multiple data sources indicating species presence (sightings and passive acoustic monitoring). We conclude that, when available, assessing species presence according to multiple sources of inference will be central to developing robust predictions to support dynamic ocean management.

## Introduction

The demand to balance the sometimes conflicting objectives of marine life protection and human uses has led to the development of dynamic ocean management (DOM) measures that rapidly change in space and time through the integration of near real-time biological, oceanographic or social data (Maxwell et al., 2015). Growing availability and access to near real-time data, particularly from remotely sensed environmental observations, combined with advanced computing power, has made this novel approach possible. Species distribution models (SDMs) based on species–environment relationships, are being increasingly operationalized with near real-time environmental data for the purpose of informing DOM to address a range of conservation threats, including marine megafauna bycatch (Howell et al., 2008; Hazen et al., 2018), entanglements (Samhouri et al., 2021) and vessel collisions (Hazen et al., 2017; Ham et al., 2021; Hausner et al., 2021). There remain some reservations, however, concerning the appropriate use of SDMs in guiding conservation or management decisions designed to mitigate human–wildlife conflict (Guisan et al., 2013; Robinson et al., 2017).

In the California Current System (CCS), vessel collisions remain a leading factor limiting the recovery of endangered Northeast Pacific blue whales (*Balaenoptera musculus*) to pre-whaling-era numbers (NOAA 2023 Stock assessment). An SDM using near real-time environmental data to predict where whales were likely located, WhaleWatch, was developed to mitigate whale-vessel collision risk (Hazen et al., 2017). An updated higher resolution version, WhaleWatch 2.0 (Abrahms et al., 2019), has become one of three data sources, alongside sightings and passive acoustic monitoring (PAM) detections, supporting the Whale Safe initiative (https://whalesafe.com/). This initiative supports the NOAA-led vessel speed reduction program in California by sharing whale data with shipping companies and monitoring their cooperation with speed recommendations during the months of peak endangered whale abundance. It has successfully helped improve voluntary vessel speed reduction cooperation (https://whalesafe.com/). However, few areas and species are as data-rich; thus, understanding the reliability of operational SDM outputs as stand-alone metrics of species presence in relation to *in situ* data sources is an important step in enabling possible future expansion of the number of species and systems that could support DOM frameworks (*e.g.*, cryptic species where sightings are rare, cost constraints).

Here we use Whale Safe, a tool designed to prevent lethal and sublethal ship strikes with whales, and the endangered blue whale as a case study to demonstrate the contribution of an SDM in the context of other available whale presence data. Our objectives were to (1) evaluate the contribution of the SDM relative to concurrent detections of blue whale presence, and (2) to compare the WhaleWatch 2.0 (blue whale SDM, hereafter) predictions with sightings and passive acoustic detections of blue whales. From this analysis, we show the correlation between the model predictions and whale detections. The value added by near real-time model predictions depends on whether species-specific *in situ* data are consistently available, meaning accessible without interruption (*e.g.*, without technological failures or seasonal limitations). When *in situ* data are opportunistic or sporadic, model predictions provide essential insights into species presence that may otherwise be unavailable (*i.e.*, *in situ* data gaps). Additionally, simultaneous contributions from multiple data sources provide inference for different components of whale presence (sightings: where/when they surface, acoustics: where/when they call and SDMs: where/when there is suitable habitat), and also have distinct but complementary limitations. For example, sightings and passive acoustic detections are prone to false negatives (whales present but not detected), while SDM predictions have uncertainties but are optimized to balance false positives and negatives. Such co-occurring sources of insight (hereafter, multiple data sources) allow for a better understanding of whale presence, as well as uninterrupted data delivery even during failure of any single data stream, supporting the long-term success of DOM.

## Methods

We used blue whale presence from each of Whale Safe’s three data sources (sightings, acoustics and SDM predictions), which were aggregated, processed and provided to us by Whale Safe. These data sources were analyzed for two regions in the CCS where Whale Safe operates: (1) the San Francisco Vessel Speed Reduction (VSR) Zone, and (2) the Santa Barbara Channel VSR Zone off Southern California, USA. Sightings data were a combination of opportunistic naturalist observations reported to Whale Alert and Spotter Pro mobile apps and monthly aerial surveys in the Santa Barbara Channel. No metric for effort was available for any of the sightings data. Acoustic detections were collected by near real-time PAM buoy systems, one off San Francisco and one in the Santa Barbara Channel (Baumgartner et al., 2019). The percentage of 15-min summary periods per day with blue whale acoustic detections was calculated by Whale Safe, and represents the number of 15-min periods with blue whale detections (robots4whales.whoi.edu) relative to the total number of monitored 15-min periods for that day. Lastly, the blue whale SDM was developed by Abrahms et al. (2019) using location data from satellite-tagged blue whales (Hazen et al., 2017). The blue whale SDM is a multimodel ensemble (https://coastwatch.pfeg.noaa.gov/projects/whalewatch2/whalewatch2_map.html), and the predictions are generated using both Generalized Additive Mixed Model and Boosted Regression Tree modeling frameworks (Abrahms et al., 2019). Ensemble models can improve model accuracy, as was seen with the approach by Abrahms et al. (2019) for this blue whale SDM. The model predicts blue whale habitat suitability, a score ranging from 0 to 1 where 0 indicates unsuitable habitat and 1 represents regions where the model predicted optimal habitat conditions. Habitat suitability predictions were based on environmental conditions hypothesized to influence blue whale habitat use, including sea surface temperature, sea surface height and isothermal layer depth from Regional Ocean Modeling Systems configured to the CCS (oceanmodeling.ucsc.edu). Habitat suitability was predicted daily (0–100%) across the CCS with a spatial resolution of 10 km × 10 km grid cells. Within the Whale Safe program, detections from each of the data sources are assigned a categorical “high,” “medium” or “low” rating based on the number of detections/sightings per day and the SDM value to qualitatively characterize the probability of whale presence for the purpose of informing voluntary speed reduction (Table 1). To visualize the contribution of the three data sources in each region, we summarized the daily records from each of the continuous data sources into monthly averages, as well as the number of days that each data stream was offline during the study period.

**Table 1.** Summary of methodology followed by Whale Safe to categorically rank blue whale presence for each of its three data sources (sightings, acoustics and a blue whale SDM). More details on each ranking can be found on the Whale Safe website (https://whalesafe.com/methodology/) Table 1. long description.

Sightings data	
Number of whales observed (daily)	Whale presence rating
0	Low
1	Medium
>1	High

Acoustic data	
Percentage of summary periods with confirmed acoustic detections (daily)	Whale presence rating
0%	Low
0–10%	Medium
≥10%	High

Blue whale SDM	
San Francisco	
Average habitat suitability across vessel speed reduction zone	Whale presence rating
<0.70	Low
≥0.70	Medium

Santa Barbara Channel	
Average habitat suitability across vessel speed reduction zone	Whale presence rating
<0.75	Low
≥0.75	Medium

A generalized linear mixed-effect model (GLMM) framework with the *glmmTMB* package in R (Brooks et al., 2017) was used to evaluate the relationships between the blue whale SDM predictions and the *in situ* sources of whale presence (sightings and acoustic data) on a daily scale when data were available. Sightings or acoustic data served as the response variable. Seasons form part of a cycle that impacts whale presence (*i.e.*, migratory behaviors), which can be described by the angular equivalent of the state of the cycle using sine and cosine functions (Bailey et al., 2013). Month was converted into two vectors using sine and cosine terms, and both terms were included as fixed effects. An interaction between the SDM predictions and region (*i.e.*, San Francisco and the Santa Barbara Channel) was also included as a fixed effect to allow for regional differences in the relationships between the habitat suitability predictions and *in situ* data. The Santa Barbara Channel served as the reference level. Year was centered and included as a random effect to account for interannual variation. A Poisson distribution with a log link function was used for the sightings model, where the response variable represented count data, while a beta distribution with a logit link function was used for the acoustics model, where the response variable represented a percentage of summary periods with acoustic detections. We allowed zero inflation to vary by region.

## Results

Blue whale sightings, acoustic detections and SDM predictions were analyzed across 846 days off San Francisco (September 2022–January 2025) and 1,579 days in the Santa Barbara Channel (September 2020–January 2025). The acoustic and SDM data went offline for a prolonged period (*n*_acoustic_ = 474 days, *n*_model_ = 170 days off San Francisco; *n*_acoustic_ = 389 days, *n*_model_ = 180 days in the Santa Barbara Channel) due to technological failures and damage sustained by the moored PAM systems. Given the opportunistic nature of the sightings data, there was no information available to account for effort or recorded days with missing visual effort. The offline time periods often differed between the acoustic and modeled data sources, meaning there was an increased likelihood that at least one data stream was active for each day of the study period.

**Figure 1.**
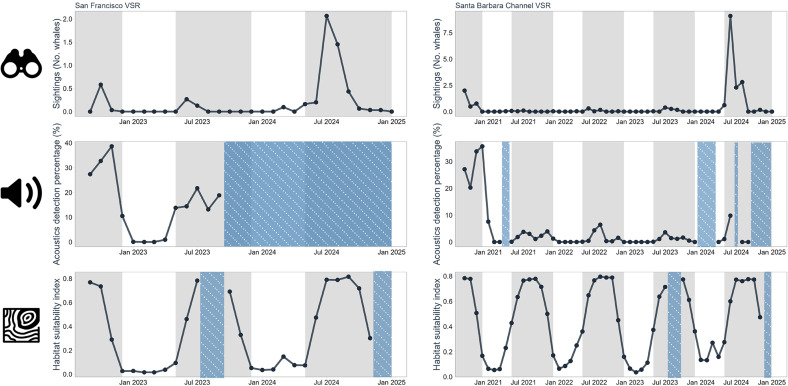
Year-month averages of blue whale sightings records (top panel), acoustic detections (middle panel), and blue whale habitat model predictions (bottom panel). Data are separated by study region, which includes San Francisco (left) and the Santa Barbara Channel (right). Grey rectangles display when the voluntary vessel speed reduction (VSR) zone was active and blue dotted rectangles indicate when a given data type was “offline”. Figure 1. long description.

There were more frequent acoustic detections of blue whales than reported sightings in both regions (Figure 1). Sighting numbers were generally very low and peaked in both regions in summer 2024. There were relatively high blue whale acoustic detections in 2020 in the Santa Barbara Channel, but they were much lower from 2021 onwards, other than briefly in mid-2024. Acoustic detections of blue whales were much higher off San Francisco in 2022–2023 than during the corresponding period in the Santa Barbara Channel (Figure 1).

The blue whale habitat suitability predictions were positively correlated with sightings data in the Santa Barbara Channel (×2.63 as many whales when habitat suitability is 1, *P* = 0.032, *n* = 1,402) (Table 2). There was also an insignificant regional difference observed between the WhaleWatch predictions and sightings data (*P* = 0.079, *n* = 2080), suggesting a weak, but positive relationship between sightings data and WhaleWatch predictions in San Francisco (×0.90 as many whales when habitat suitability is 1). A positive relationship was also observed between the WhaleWatch habitat suitability predictions and acoustics data in the Santa Barbara Channel (×5.34 as many summary periods with acoustic detections when habitat suitability is 1, *P* < 0.0001, *n* = 1,089). The relationship between the WhaleWatch predictions and acoustic detections significantly differed between the two regions (*P* < 0.0001, *n* = 1,379). Acoustic detections had a stronger positive relationship with predicted habitat suitability in San Francisco (×25.69 as many summary periods with acoustic detections when habitat suitability is 1, *P* = 0.0079, *n* = 290).

**Table 2.** Results from the acoustics and sightings data generalized linear mixed models (GLMMs) Table 2. long description.

Parameter	Coefficient estimate	Confidence interval	*P*
Sightings data model			
Intercept	0.428	0.126–1.453	0.174
WhaleWatch	2.631	1.086–6.375	**0.032**
Region – SF	1.399	0.565–3.465	0.468
Month (sin)	0.536	0.368–0.781	**0.001**
Month (cos)	0.287	0.193–0.410	**<0.0001**
WhaleWatch: Region – SF	0.341	0.103–1.133	0.079
Acoustic data model			
Intercept	44.356	33.458–55.825	0.335
WhaleWatch	5.340	2.081–13.024	**<0.0001**
Region – SF	31.747	22.294–42.989	**0.002**
Month (sin)	21.580	14.801–30.358	**<0.0001**
Month (cos)	50.548	44.932–56.149	0.849
WhaleWatch: Region – SF	85.974	75.211–92.528	**<0.0001**

Variables with *P* < 0.05 are bolded. The Santa Barbara Channel served as the reference level for the region parameter. Coefficient estimates have been converted back to the response scale, and the 95% confidence interval is provided. The WhaleWatch parameter represents the habitat suitability prediction.

## Discussion and conclusions

The rapid changes taking place in the marine environment due to unprecedented environmental change will require dynamic management approaches that can respond to such change while balancing ecological, social and economic priorities. DOM approaches can help to meet these demands, although they have large data requirements that can serve as a barrier to their success and implementation (Hobday et al., 2013; Lewison et al., 2015; Maxwell et al., 2015; Smith et al., 2021). Thus, identifying possible solutions to such barriers may be key to the broader application of DOM. Our results indicate that SDM outputs can provide valuable contributions alongside *in situ* monitoring for detecting whale presence (sightings records and PAM). While the multiple data sources available for Whale Safe may serve as the “gold standard” given the benefits of co-occurring sources of insight, the correlations between the independent *in situ* data sources and the blue whale SDM suggest that SDMs have potential to serve as indicators for the probability of species presence, *via* habitat suitability predictions, when other data sources are unavailable (*e.g.*, technological failures, seasonal or cost limitations). San Francisco and the area around the Santa Barbara Channel serve as important habitat for blue whales (Irvine et al., 2014), yet additional research should also be completed to investigate the different results observed between these regions that could be attributed to a range of factors (*e.g.*, interannual variability of whale presence in the two regions, differences in sightings effort, changes in vessel traffic and ambient sound levels making it easier/harder to detect whale calls and the number of days the PAM buoys were online/offline). Ideally, stronger relationships between SDM predictions and the sightings data would be observed for both regions before relying on SDMs alone to guide conservation and management decisions. Together, however, these results support the use of SDMs to address some of the data requirements for DOM initiatives, expanding the overall accessibility of this climate-ready management approach.

DOM programs may rely on consistent, near real-time access to species presence data to guide the regulation of various ocean resource-user groups (*e.g.*, shipping companies and fisheries (Lewison et al., 2015; Maxwell et al., 2015; Barlow and Torres, 2021). Data sources can include fisheries-dependent logbook records, PAM, SDM habitat suitability predictions and bio-logging or bio-telemetry detections, among others (Hazen et al., 2018; Oestreich et al., 2020; Pons et al., 2022; Schwartz-Belkin and Portman, 2023). Each data source can be used to support DOM frameworks, though each comes with their own unique set of drawbacks (*e.g.*, technological failures, cost and seasonally dependent biases), such that all three sources together in a data fusion framework, such as Whale Safe, may provide a more complete picture than any data source alone (Gilman et al., 2006; Hobday et al., 2013; Maxwell et al., 2015; Thorne et al., 2019).

Acoustic recordings rely on whale vocalizations (*e.g.*, demographically and seasonally dependent behaviors) to detect presence, while sightings rely on calm waters, good visibility, sampling effort and surfacing animals. As such, acoustics and sightings data are also prone to false negatives. Additionally, documented effort may be unavailable when using community-based sightings, as is the case in the present study, making records of species absence and relationships between sightings and modeled data difficult to interpret. A notable benefit of *in situ* data, that is not captured by the SDM, is that *in situ* data provide important insights into species-specific responses to extreme ocean conditions that may not be captured through models informed by historical data. Model predictions highlight where animals are most likely to occur based on the historical data used to train the model, but anomalous ocean conditions and the ephemeral nature of prey aggregations may result in a mismatch between model predictions and actual whale presence (Santini et al., 2021; Brown and Puschendorf, 2025). In these cases, *in situ* data provide valuable insight into potentially unexpected patterns of species presence.

Simultaneous access to multiple data sources can compensate for each dataset’s respective limitations and provide continuity during periods when some data sources are offline. The multiple data sources used to inform Whale Safe offer a number of strengths, including robust spatiotemporal coverage and multiple forms of inference for whale presence (Hausner et al., 2021). However, when multiple data sources are unavailable, as is the case for many regions and species, our results suggest that SDM outputs can fill an important real-time species presence data gap. SDMs have been presented as a valuable tool for guiding DOM approaches, and our correlation results indicate that these modeled outputs were indeed operationally reliable and could be used to assess the probability of species presence *via* habitat suitability predictions when other data sources fail or are unavailable (Hazen et al., 2013; Brodie et al., 2015; Scales et al., 2017). Ecologically realistic SDM predictions have been recommended for marine spatial planning (Milles et al., 2025), anticipating marine heatwave impacts on fisheries (Farchadi et al., 2024), and supporting marine conservation and management more broadly (Braun et al., 2023).

While the use of SDM outputs for guiding DOM offers many benefits (*e.g.*, broad spatial coverage, multispecies and fine-scale resolution), the validation and maintenance of these models and tools over long periods of time may only be possible with continuous support and near real-time environmental data availability (Leidner and Buchanan, 2018). Academic-sponsored projects dependent on grant cycles may not be able to sustain such programs long term (Welch et al., 2019; Barlow and Torres, 2021). The automated operationalization of WhaleWatch, in addition to NOAA’s continuous support and maintenance after the initial development funding from NASA, has underpinned its longevity. SDMs can require large amounts of species distribution data for their development and validation (Wisz et al., 2008), although approaches for more sparse datasets are being developed (*e.g.*, see Welch et al., 2019). They also need periodic reassessment and potential refitting as the historical relationships identified between species and environmental conditions may alter over time with climate change (Elith and Leathwick, 2009). This may be especially important given potential species redistributions due to anticipated ocean variability. Such factors will be critical to incorporate into the design of future SDM tools to support the broad application of DOM (Welch et al., 2019). DOM may play a core role in climate adaptation strategies, thus identifying the features and mechanisms that contribute to the success of these programs will be key to its efficacy into the future.

## Data Availability

The dataset supporting the results of the study can be made available on request.

## Long descriptions

### Table 1. Long description

The table is divided into three main sections:

1. Sightings data:

- 0 whales observed daily is rated Low.

- 1 whale observed daily is rated Medium.

- Greater than 1 whale observed daily is rated High.

2. Acoustic data:

- 0 percent of summary periods with confirmed acoustic detections daily is rated Low.

- 0 to 10 percent is rated Medium.

- Greater than or equal to 10 percent is rated High.

3. Blue whale S D M (Species Distribution Model):

- For San Francisco: Average habitat suitability across vessel speed reduction zone less than 0.70 is rated Low; greater than or equal to 0.70 is rated Medium.

- For Santa Barbara Channel: Average habitat suitability across vessel speed reduction zone less than 0.75 is rated Low; greater than or equal to 0.75 is rated Medium.

Navigate back to Table 1..

### Figure 1. Long description

A six-panel grid of line graphs. The left column is titled San Francisco V S R and the right column is titled Santa Barbara Channel V S R.

Top Row (Visual Sightings): The y-axis is Sightings (No. whales). San Francisco shows a major peak of 2.0 in July 2024. Santa Barbara shows a major peak of nearly 10.0 in July 2024. Both show low activity in 2023.

Middle Row (Acoustic Detections): The y-axis is Acoustics detection percentage (%). San Francisco shows peaks around 40% in early 2023 and 20% in mid-2023, followed by a large blue hatched area from late 2023 to 2025. Santa Barbara shows a peak of 35% in early 2021, with intermittent blue hatched zones in 2021 and 2024.

Bottom Row (Habitat Suitability): The y-axis is Habitat suitability index from 0 to 0.8. Both regions show a cyclical, wave-like pattern. San Francisco has peaks in early 2023, mid-2023, and mid-2024. Santa Barbara shows more frequent oscillations with peaks in early 2021, mid-2021, mid-2022, mid-2023, and mid-2024.

Icons on the far left indicate the data source: binoculars for sightings, a speaker for acoustics, and a topographic map for habitat suitability. Vertical gray shaded bars indicate seasonal periods across all graphs.

Navigate back to Figure 1..

### Table 2. Long description

The table is divided into two main sections.

Section 1: Sightings data model.

- Intercept: Coefficient 0.428, Confidence interval 0.126–1.453, P 0.174.

- WhaleWatch: Coefficient 2.631, Confidence interval 1.086–6.375, P 0.0321.

- Region – S F: Coefficient 1.399, Confidence interval 0.565–3.465, P 0.468.

- Month sin: Coefficient 0.536, Confidence interval 0.368–0.781, P 0.00115.

- Month cos: Coefficient 0.287, Confidence interval 0.193–0.410, P less than 0.0001.

- WhaleWatch or Region – S F: Coefficient 0.341, Confidence interval 0.103–1.133, P 0.0792.

Section 2: Acoustic data model.

- Intercept: Coefficient 44.356, Confidence interval 33.458–55.825, P 0.335.

- WhaleWatch: Coefficient 5.340, Confidence interval 2.081–13.024, P less than 0.0001.

- Region – S F: Coefficient 31.747, Confidence interval 22.294–42.989, P 0.00190.

- Month sin: Coefficient 21.580, Confidence interval 14.801–30.358, P less than 0.0001.

- Month cos: Coefficient 50.548, Confidence interval 44.932–56.149, P 0.849.

- WhaleWatch or Region – S F: Coefficient 85.974, Confidence interval 75.211–92.528, P less than 0.0001.

Note: P-values less than 0.05 are considered significant. The Santa Barbara Channel served as the reference level for the region parameter.

Navigate back to Table 2..
