# Supplementary material for: The role of species distribution models and multiple data sources in implementing dynamic ocean management
Source: Camb Prism Coast Futur. 2026 Jun 24;4:e20. doi: 10.1017/cft.2026.10041 (PMC13419462; doi:10.1017/cft.2026.10041)
Supplement: Peer review history

# Reviewer report: Review: The role of species distribution models and multiple data sources in implementing dynamic ocean management — R0/PR2

**DOI:** 10.1017/cft.2026.10041.pr2

Summary:

This manuscript is directed towards conveying how species distribution models (SDM) can support dynamic ocean management efforts of species of concern through a real-world case

study—blue whales in the California Current System off San Francisco and Santa Barbara. To

achieve these results, the authors leverage model predictions developed by Abrahms et al. (2019,

Diversity and Distributions) in the context of two other data streams, sightings and acoustics

data.

The authors demonstrate that the SDM predictions were correlated with sightings and

acoustics data with two tests, Spearman and Kendall correlation coefficients in both regions

although correlation was stronger in San Francisco compared to Santa Barbara. Together, the

authors have compiled and presented a manuscript that illuminates the utility of incorporating

SDMs into the portfolio data streams supporting dynamic ocean management, such as when in-

situ data streams may be unavailable. Combined, findings have clear applications that can assist

regional management. While the authors alluded to the shortcomings inherent with in-situ data

streams, greater focus on the shortcomings of SDM predictions would assist in demonstrating a

more holistic interpretation of observed results. The results are informative and I recommend

publication after major revision.

Major issues:

The greatest weakness of the manuscript is its characterization of the seeming objectivity of the SDMs

compared to the sightings and acoustics data streams. There is no doubt that aggregating multiple

streams of data is necessary to provide more complete analysis for dynamic ocean management (i rely on SDMs for my work as well!)

Nevertheless, while there is an indication that SDMs can be optimized compared to other data

streams on lines 80 and 185–186, there is no elaboration on how the model used to drive Whale

Watch 2.0 was optimized. Indeed, details of the model can be read more extensively in Abrahms

et al. (2019, Diversity and Distributions) but the present manuscript would benefit from a brief

overview on what was optimized in the underlying model. It is also worth mentioning that while

it is possible to account for underlying biases in SDMs, there will always be some residual level

of bias. For example, the model driving Whale Watch 2.0 is a boosted regression tree and is

informed by electronic tag data, yet random effects are not possible to include in a boosted

regression tree. If actions were taken to reduce the spatial autocorrelation of data driving the

SDM referenced here, the authors should mention such. It would also bolster the authors

argument that SDMs are robust tools to support dynamic ocean management by giving examples

of ecologically realistic predictions from boosted regression tree SDMs in the literature (see

Braun et al. 2023, Ecological Applications; Farchadi et al. 2024, Fish and Fisheries; Milles et al.

2025, Marine Ecology Progress Series).

Along similar lines, the authors mention that the present SDM was more correlated with the

other data streams in San Francisco compared to Santa Barbara. As a reader, I would appreciate

further discussion into why this may be; where were the majority of underlying electronic tag

data located? While these data again are available in Abrahm et al. (2019, Diversity and

Distributions), it would improve the nuance and reduce potential overoptimism of the model

performance compared to greater pessimism of the other data streams (this is not to say one data

stream is more robust; there needs to be more discussion of the model performance to “level the

playing field”). These findings may also allude to the importance of the domain and scale in

developing and predicting SDMs. The California Current System is particularly dynamic;

therefore, there may be regions in which the model performs better than others. It may be

worthwhile to mention the ability of the model to capture (or fail to) the relation between blue

whales and predicted suitable habitat compared to the other data streams when analyzing the

correlation values stated in the results.

Minor Comments:

- On line 49, are you referencing the International Union for Conservation of Nature of

U.S. Endangered Species Act when referencing “endangered”?

- On line 127, there is reference to days where SDM predictions were unavailable due to

the model going offline. It is unclear to me if the model is being predicted to

environmental data recorded by PAMs. And if the PAM went offline and the model is

being predicted to PAM arrays, why would the acoustic stream have more offline days

compared to the SDM? I think it is likely a clarity issue.

I thank the authors for an interesting read and thank the editor for the opportunity to review.

References:

Braun, C.D. et al. (2023) “Building use‐inspired species distribution models: Using multiple

data types to examine and improve model performance,” Ecological Applications, 33(6), p.

e2893. Available at: https://doi.org/10.1002/eap.2893.

Farchadi, N. et al. (2024) “Marine heatwaves redistribute pelagic fishing fleets,” Fish and

Fisheries, n/a(n/a). Available at: https://doi.org/10.1111/faf.12828.

Milles, H.M. et al. (2025) “Characterizing seasonal whale shark habitat in the western North

Atlantic,” Marine Ecology Progress Series, 766, pp. 91–106. Available at:

https://doi.org/10.3354/meps14906.

# Reviewer report: Review: The role of species distribution models and multiple data sources in implementing dynamic ocean management — R0/PR3

**DOI:** 10.1017/cft.2026.10041.pr3

The work is interesting and could be a necessary contribution to understanding how different sources of information can be useful in modulating species distributions, specifically marine species such as cetaceans. Although the work raises this need and presents an analysis with whale sighting data in different formats, it is actually unclear in several respects, so I suggest that these issues be carefully addressed before it can be accepted for publication.

The article is not consistent with the terms most commonly used in SDM, so it would be advisable to review the publication Melo-Merino et al. 2020 Ecol. Model. (https://doi.org/10.1016/j.ecolmodel.2019.108837) for a more comprehensive reference on these issues. In this publication, you will find the need to clarify several aspects that are relevant, on the one hand, to the concepts and, on the other, to the implementation of the tools. For example, the use of the term habitat-based model may be appropriate but at the same time confusing if it is not made clear why this term and not another is used, given the objective of moderation.

Another aspect that may be confusing from the title’s approach could be what Dynamic means. Although it is clear that they are talking about dynamic ocean management, this can be very vague and could even be confused (or confuse), for example, with dynamic SDM modeling approaches (e.g., see Dobson et al. 2023 MEE; https://doi.org/10.1111/2041-210X.14101). In other words, this distinction and objective need to be clarified in greater depth. This same idea about the need to be more specific is scattered throughout the text, with other examples of terms including the following: additional/multiple data streams (in the impact statement or on line 187); near real-time habitat; multiple data sources and data redundancy; among the most important.

Another aspect that may be important is the relationship between this work/analysis and what is known as time-specific niche modeling. I bring this up because it specifies this idea of near real-time data (gap) and therefore opens up the possibility of thinking about this concept of making time-specific models, which, although it is something that is only just being explored, already has a perhaps more direct meaning with what is being proposed.

In general terms, my opinion is that the work offers much more than can be understood after reading it, and it really detracts from leaving a clear notion of how this can be operated (i.e., data + concepts) in a clear and forceful manner.

Hence my suggestion that it be carefully reviewed and addressed by pointing out and linking the concepts to existing literature. I am attaching a file for reference to my comments here.

# Editor report: Recommendation: The role of species distribution models and multiple data sources in implementing dynamic ocean management — R0/PR4

**DOI:** 10.1017/cft.2026.10041.pr4

**Affiliations:** The University of British Columbia, Canada

Dear authors,

We have now received two evaluations of your manuscript. Although we had initially sought an additional review, we believe the two reports obtained provide sufficient and valuable feedback for the manuscript.

Both reviewers suggested major revisions so I am therefore returning the manuscript to you with the expectation that you can consider their comments, which I agree will strongly improve the paper. Please pay particular attention to improving the clarity of the overall narrative and the methods section. Both reviewers have provided references that may help guide your revisions.

Thank you for your work and for your patience throughout the review process.

# Editor report: Decision: The role of species distribution models and multiple data sources in implementing dynamic ocean management — R0/PR5

**DOI:** 10.1017/cft.2026.10041.pr5

**Affiliations:** Geography, Cambridge University, United Kingdom of Great Britain and Northern Ireland

No accompanying comment.

# Reviewer report: Review: The role of species distribution models and multiple data sources in implementing dynamic ocean management — R1/PR7

**DOI:** 10.1017/cft.2026.10041.pr7

After reviewing the manuscript for a second time, I realized that the presented analysis and text do not adequately account for (1) non-random missing data and (2) unaddressed temporal dependence, both of which may bias the strength and significance of the reported correlations. Moreover, (3) there is no clear explanation of how the SDMs were constructed in terms of environmental predictors. The link provided in the text does not appear to work, and there is insufficient detail regarding the variables or factors included in the models. In addition, there is no clear delineation of the species’ accessibility area (see Barve et al. 2011, Ecological Modelling), which is crucial for many modeling algorithms given its strong influence on model performance and statistical significance. As currently presented, there is no clear justification for what “suitability” represents in the modeling framework. I therefore encourage the authors to carefully consider these comments and strengthen both the analysis and the manuscript accordingly. Overall, these issues should ideally be addressed through additional analyses to ensure the robustness of the results or at least a really robust justification, but preferred the reanalysis of the data.

Regarding the first general comment, the analyses only include days when the monitoring system was online. Because offline periods may coincide with environmental conditions that also influence whale presence or detectability, the missing data may not be random. This could introduce selection bias, meaning that the reported correlations may not represent patterns across the full study period. The authors could evaluate whether online/offline status is systematically related to environmental or detection variables and, if necessary, apply statistical adjustments to account for potential bias.

Regarding the second point, the correlation tests do not account for temporal autocorrelation or seasonality in the daily time series. Since daily ecological data are typically temporally structured, treating observations as independent may inflate statistical significance. As a result, the reported p-values may overstate the strength of the associations. The authors could reanalyze the data using time-series approaches that account for autocorrelation and seasonal patterns, or implement a more comprehensive model-based framework.

# Reviewer report: Review: The role of species distribution models and multiple data sources in implementing dynamic ocean management — R1/PR8

**DOI:** 10.1017/cft.2026.10041.pr8

I thank the authors for an interesting read and for being responsive to my feedback. I recommend publication after minor revision.

The authors have resolved my previous feedback concerning the need for greater context surrounding the models that feed into Whale Safe and have added the details sufficient for a perspective in Cambridge Prisms: Coastal Futures.

The authors have also sufficiently revised the manuscript to acknowledge the differences in correlation values among regions and allude to the gap in knowledge that should be explored as DOM efforts are pursued.

Minor comment

The manuscript sometimes alludes that the SDM predictions are indicative of species presence

(80–88; 163–165; 209). While acoustics and visual sightings directly identify whale presence,

SDMs do not. I recommend reframing that the SDM predicted values are not directly linked to

species presence; rather, the model predicts optimal habitat conditions which may incur greater

probable presence. I encourage the authors to review the manuscript for additional examples I

may have missed.

# Editor report: Recommendation: The role of species distribution models and multiple data sources in implementing dynamic ocean management — R1/PR9

**DOI:** 10.1017/cft.2026.10041.pr9

**Affiliations:** The University of British Columbia, Canada

Dear Authors, 

As you can see one of the reviewers has further questions regarding the methodology of the SDM employed while the other one is satisfied. I am returning the manuscript to you with the hopes that you can further address the concerns of the reviewer. While I don’t expect you to run all the analysis required by the reviewer, I would like to see strong justifications for those not directly addressed by extra analysis. 

Many thanks

# Editor report: Decision: The role of species distribution models and multiple data sources in implementing dynamic ocean management — R1/PR10

**DOI:** 10.1017/cft.2026.10041.pr10

**Affiliations:** Geography, Cambridge University, United Kingdom of Great Britain and Northern Ireland

No accompanying comment.

# Editor report: Recommendation: The role of species distribution models and multiple data sources in implementing dynamic ocean management — R2/PR12

**DOI:** 10.1017/cft.2026.10041.pr12

**Affiliations:** The University of British Columbia, Canada

Dear Authors, 

I want to start by personally apologize for the length of the revision process and thank you for your patience. I am pleased with the revisions you have made and delighted to let you know your manuscript has now been accepted for publication.

# Editor report: Decision: The role of species distribution models and multiple data sources in implementing dynamic ocean management — R2/PR13

**DOI:** 10.1017/cft.2026.10041.pr13

**Affiliations:** Geography, Cambridge University, United Kingdom of Great Britain and Northern Ireland

No accompanying comment.
